# An LC-MS-Based Molecular Networking Proof-of-Concept for Revealing Ecological Functional Dynamics in Nylsvley Ramsar Wetland Waters

**DOI:** 10.1007/s00128-026-04258-3

**Published:** 2026-05-18

**Authors:** Florence Mazwi Murungweni, Nakisani Babra Moyo, Farai Dondofema, Ntakadzeni Edwin Madala

**Affiliations:** 1 Department of Geography and Environment, University of Venda, P.O. Bag X5050, Thohoyandou, Limpopo Province South Africa; 2 Department of Food Science and Technology, University of Venda, P.O. Bag X5050, Thohoyandou, Limpopo Province South Africa; 3 Centre of Excellence in Mass Spectrometry for Southern Africa (CEMSSA), University of Venda, P.O. Bag X5050, Thohoyandou, Limpopo Province South Africa

**Keywords:** Ecosystem, Mass spectrometry, Molecular networking, Organic pollutants, Wetlands

## Abstract

Wetlands are unique ecological niches that support highly specialized ecosystems, yet their functioning is strongly influenced by surrounding human activities. Increasing settlement around Nylsvley, a Ramsar-designated wetland has resulted in observable ecological changes. This study presents a comprehensive chemical profiling of water from two sites; Jacana Hide Site (JHS) and Site 3 (S3) sampled during dry and wet seasons. Untargeted LC-MS combined with molecular networking revealed clear chemical differences between sites, with JHS showing greater chemical variability than S3. Many detected compounds could not be annotated due to current database limitations. Among the identified compounds, several anthropogenic contaminants were detected, including plastic-associated fatty acid amides (erucamide and oleamide), phthalates and a UV-filter 2-hydroxy-4-methoxybenzophenone, which may pose ecological risks to aquatic organisms. In addition, alkaloids commonly associated with tobacco and opium use were also observed, suggesting potential wastewater-derived inputs into the wetland system. Molecular networking also showed a notable shift in the chemical composition of the investigated sites before and after rainfall. Overall, this work serves as a proof of concept, providing an initial assessment of the chemical status of the Nylsvley wetland and highlighting potential toxicological risks associated with emerging contaminants.

**Supplementary Information:**

The online version contains supplementary material available at 10.1007/s00128-026-04258-3.

## Introduction

Wetlands are ecosystems found between aquatic and terrestrial environments, and they are biologically diverse ecosystems, globally, as they are rich in flora and fauna (Cherry 2011; Mitsch and Gosselink 2015). They perform essential biogeochemical and hydrological functions, such as carbon sink, flood mitigation, water purification, recharge of ground aquifers, among others (Cherry 2011; Whitcomb et al. 2009). Despite the crucial roles provided by wetlands, human development within, around, or upstream of wetlands disturbs the natural functioning of these systems, making it necessary to develop and utilise technologies that help better evaluate the impacts, provide protection, and improve the management of these systems.

Previous studies conducted in the Nylsvley Nature Reserve (NNR), a Ramsar site of international importance, have revealed poor water quality and a drastic decline of 80-90% in aquatic bird species visiting the wetland (Greenfield et al. 2007, 2012). Similarly, the frog population, once comprising nineteen species, has declined significantly, although the exact number is unknown, as few individuals can now be heard calling after rainfall, suggesting reduced abundance (Greenfield et al. 2007, 2012). These ecological declines point to the adverse effects of upstream human activities on the NNR, which lies downstream of Modimolle town. However, the specific pollutants responsible for this remain largely uncharacterized. Therefore, there is an urgent need to monitor the chemistry of wetlands to better understand upstream impacts of human activities, and to explore appropriate monitoring tools to mitigate wetland degradation caused by pollution, vegetation loss, and habitat destruction, all of which contribute to biodiversity loss.

With the advent of high-resolution and high-throughput mass spectrometry instrumentation, such as hybrid quadrupole time of flight (qTOF) systems capable of performing simultaneous MS/MS data acquisition through data-dependent acquisition (DDA) modes, it has become possible to generate high-quality datasets for a range of applications, including environmental monitoring (Petras et al. 2021; Stincone et al. 2023). Molecular networking on the Global Natural Product Social Molecular Networking (GNPS) platform has further reshaped mass spectrometry data analysis by enabling the visualisation of the full chemical diversity within complex samples and facilitating powerful qualitative comparisons between groups (Ramabulana et al. 2023). To the best of our knowledge, this is the first study to use mass spectrometry for untargeted characterisation of chemical composition variation within a natural wetland ecosystem. Although previous studies have employed mass spectrometry in wetland research, these were largely focused on constructed or makeshift wetland systems and primarily targeted specific chemical entities (Duncan et al. 2020). In the present study, feature-based molecular networking was used to visualise differences in water chemistry before and after annual rainfall. The findings may support efforts to restore degraded wetlands, aligning with the goals of the United Nations Decade on Ecosystem Restoration (Mekouar 2020).

## Materials and Methods

### Chemicals and Reagents

LC-MS grade water and methanol (> 99.9%) were purchased from Romil SpS (Cambridge, UK). Analytical grade formic acid (≥ 98%) was bought from Sigma-Aldrich (Johannesburg, South Africa).

### Instrumentation

A liquid chromatography-quadrupole time-of-flight mass spectrometer (LC-MS-9030 qTOF, Shimadzu Corporation, Kyoto, Japan) was used for the chemical analysis of water samples. Separation was achieved on a Evosphere C_18_ column (100 × 2.1 mm, 1.7 µm) maintained at 55°C. The water samples (3 µL) were injected into the UHPLC-qTOF-MS following a modified method described by Moyo et al. (2023). Analytes were resolved using a binary mobile-phase gradient at a flow rate of 0.3 mL/min. Mobile phase A consisted of 0.1% (*v/v*) formic acid in ultrahigh-purity water, while mobile phase B contained 0.1% (*v/v*) formic acid in methanol. The elution gradient programme was as follows: 5% B from 0 to 3 min (held until 8 min), increased to 40% at 8 min, then to 95% between 23 and 25 min. At 27 min, the gradient returned to 5% B and was held until 30 min for column re-equilibration. Mass spectral data were acquired using an electrospray ionisation (ESI) source operated in positive-ion mode. Instrument parameters were set to, interface voltage 4.0 kV, interface temperature 300 °C, nebulising and drying gas flow 3 L/min, heat-block temperature 400°C, DL temperature 280 °C, detector voltage 1.8 kV, and flight-tube temperature 42 °C. Sodium iodide clusters were used as a calibrant to ensure high mass accuracy. MS^1^ and MS^2^ data were collected simultaneously using DDA mode across an *m/z* range of 100–1000 Da, with an intensity threshold above 3000. MS^2^ fragmentation was performed using argon as the collision gas at 30 eV. A portable multi-parameter probe (PCTestr 35, Eutech/Oakton Instruments, Singapore) was used to measure physico-chemical properties of water. Samples were dried using a freeze dryer (BTP-3ES0VX, SSP Scientific, England) prior extraction. A water bath sonicator (Scientech, Model 702) was used to sonicate reconstituted samples.

#### Study Area

The Nylsvley Wetland, located in the Nylsvley Nature Reserve, which is situated in the Waterberg region of the South African province of Limpopo (Tshimomola 2017) (Fig. S1). The wetland is surrounded by wide savanna forests and consists of large beds and grassy plants. The wetland is situated above sea level between 1080 and 1155 m. With a dry season from April to September and a wet season from October to March, the Nylsvley region receives about 620 mm of precipitation annually (Fig. S2). Most of its rainfall occurs in January, with June seeing the least amount of precipitation (Fig. S3). The average daytime temperature ranges from 20.5 °C in June to 28.9 °C in midsummer (Fig. S4). The study was carried out from two selected sites (i.e. Jacana Hide Site (JHS) and Site 3 (S3)) along the Nylsvley Wetland in December 2024 and May 2025 (Fig. S1).

#### Collection of Samples

Sampling at both the Jacana hide and Site 3 were randomly selected along a perpendicular gradient to represent the wetland (i.e. two edges). Water sampling took place in May 2024 and May 2025. Sampling was conducted during the dry season at depths below ≈approximately 61 cm. During each sampling event, at each site, environmental parameters such as pH, conductivity (μS cm^−1^), total dissolved solids (ppm), salinity (ppm), resistivity (Ω), oxygen reduction potential (mV), and temperature (°C) (*n* = 2 per site per each sampling event) were measured by immersing a portable multi-parameter probe into the water and results recorded in situ at each sampling site. For each site, at least 2-3 L of water was collected from the surface, to minimize agitation and thus target dissolved chemicals rather than sediment-associated compounds. Sampling was conducted in the morning (9:00-12:00 am) to avoid peak anthropogenic activity within the park.

#### Sample Preparation

Water samples (100 mL) in 3 replicates for each sample were freeze dried to completion and the residue was reconstituted in 5 mL of 50% aqueous methanol followed by sonication on a water bath sonicator. The samples were then filtered through a 0.22 µm nylon filters.

#### *Molecular Networking and *In Silico* Fragmentation Pattern Prediction*

Feature-based molecular networking was performed on the GNPS2 platform (https://gnps2.org) using the mgf file and feature table generated in MS-DIAL version 4.7. Precursor and MS/MS fragment ion mass tolerances were set to 0.02 Da for molecular network generation. Spectral similarity was assessed using a cosine score cutoff of 0.6, with a minimum of four matched fragments required for a connection in the network. The resulting molecular network was visualised in Cytoscape (Aron et al. 2020). SIRIUS 6.1.0 was employed to predict fragmentation patterns for compounds of interest using the mgf data (Dührkop et al. 2019). For molecular formula determination, the positive ionisation mode was selected, the MS/MS isotope scorer was set to “score,” and allowed elements included C, H, N, P, and O. Structural database searches were conducted against GNPS, HMDB, Knapsack, COCONUT, ChEBI, and Natural Products databases. Compound class prediction was carried out using CANOPUS.

## Results and Discussion

### Identified Compounds in Wetland Water Samples Using the UHPLC-qTOF-MS and Molecular Networking

**Fig. 1:**
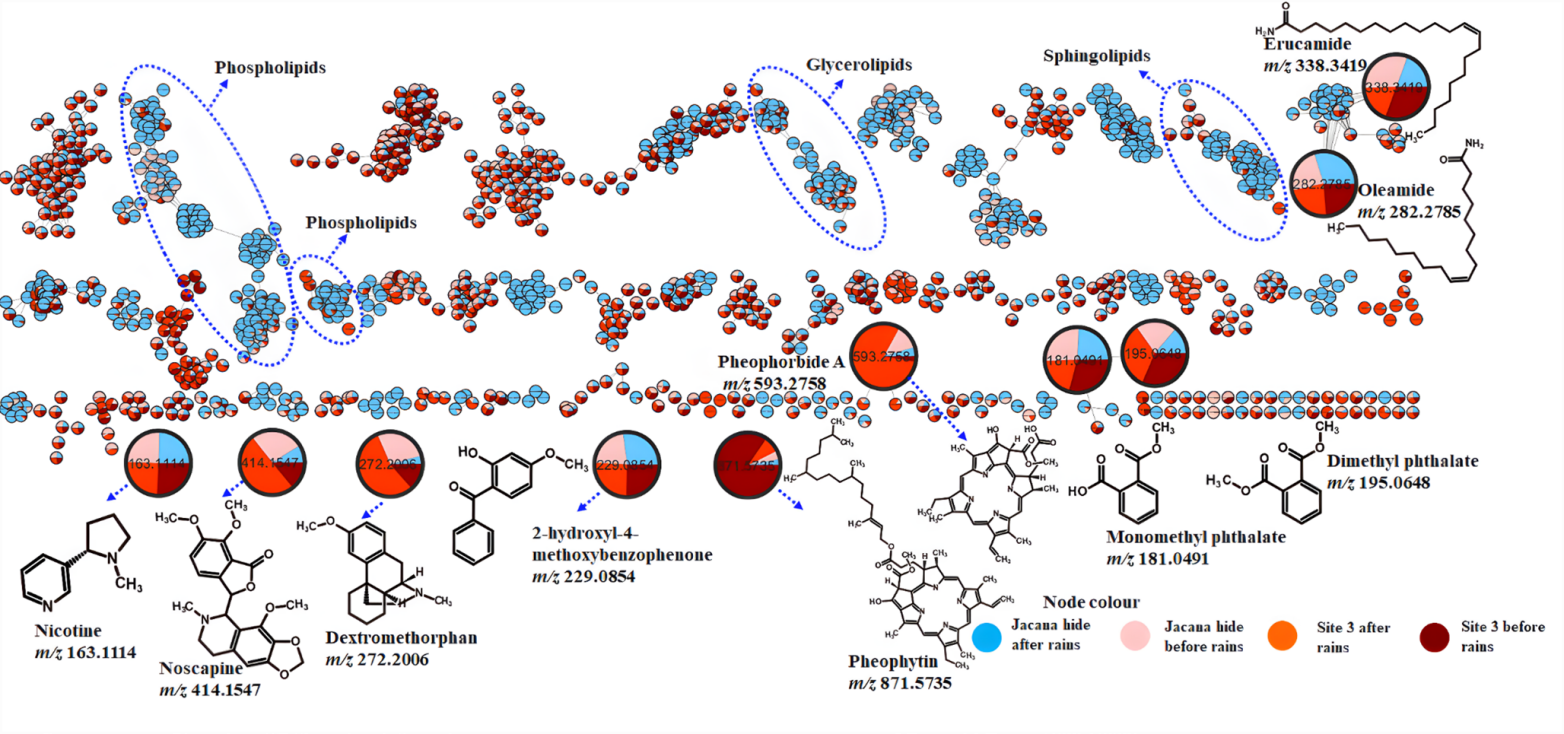
UHPLC-qTOF-MS based molecular network depicting the chemical landscape of wetland water samples. Dereplicated compounds predominantly fall within the lipid superclass. Putatively identified compounds are represented by enlarged nodes within the network

There is overwhelming evidence showing that the functionality of wetlands depends on environmental conditions and that variations in the physico-chemical characteristics of a wetland are strongly influenced by seasonal dynamics. Weather patterns such as rainfall are also expected to induce notable changes within wetland systems. In this study, an LC-qTOF-MS was used to profile water samples collected from two sites within the Nylsvley Nature Reserve (NNR), a Ramsar wetland, before and after the annual rainfall. Several identifiable compounds were detected, as shown in Fig. 1 and Table 1. All the identified compounds were observed in both sites before and after the rain. The average peak areas of the putatively identified compounds are shown in Table S1, which varied with seasons and sites, as also shown in Fig. 1. Lipids dominated the molecular network clusters, as shown in Fig. 1, indicating their prevalence in these wetlands. Notably, compounds commonly used in pharmaceutical and personal care products were detected, including an alkaloid, noscapine (*m/z* 414.1549) and dextromethorphan (*m/z* 272.2007) (Fig. 1 and Table 1). Their presence likely stems from runoff or wastewater from surrounding residential areas. Another detected alkaloid, nicotine (*m/z* 163.1114), likely reflects tobacco-related activities in surrounding areas. Nicotine is a recreational drug known for its addictive properties. Other anthropogenic compounds included fatty acid amides such as erucamide (*m/z* 338.3419) and oleamide (*m/z* 282.2785), both used as anti-sticking agents in plastics, as well as dimethyl phthalate (*m/z* 195.0648) and its metabolite monomethyl phthalate (*m/z* 181.0491), known endocrine disruptors used across the plastic, coating, and cosmetics industries. In addition to anthropogenic pollutants, natural chlorophyll derivatives, including, pheophorbide A (*m/z* 593.2758) and pheophytin A (*m/z* 871.5735), were also identified using SIRIUS, likely originating from macrophytes or algae. Moreover, the UV-filter 2-hydroxy-4-methoxybenzophenone (*m/z* 229.0854), commonly used in sunscreens and other cosmetics, was also detected. This diverse mixture of compounds, spanning from natural products, pharmaceuticals, and industrial additives, further highlights the chemical complexity of wetland systems.

**Table 1:** Putative identification of compounds in water samples from wetlands using the UHPLC-q-TOF-MS, feature-based molecular networking and SIRIUS

#	rt (min)	Compound name	Molecular ion mass	Molecular ion formula	Fragment ions	Class
1	8.77	Noscapine	414.1549	C_22_H_23_NO_7_	353, 323, 220, 205	Benzylisoquinoline alkaloid (Opium alkaloid)
2	20.03	Nicotine	163.1114	C_11_H_14_N_2_	117, 107, 105, 103	Pyridine-pyrrolidine alkaloid (tobacco alkaloid)
3	9.73	Dextromethorphan	272.2007	C_18_H_25_NO	215, 213, 171, 159, 147	Morphinan alkaloid (synthetic opioid derivative)
4	24.19	Erucamide	338.3419	C_22_H_43_NO	177, 153, 149, 135, 121, 111, 109, 107	Fatty acid amide
5	21.06	Oleamide	282.2785	C_18_H_35_NO	121, 111, 107	Fatty acid amide
6	18.55	Monomethyl phthalate	181.0491	C_9_H_8_O_4_	149, 135, 133, 121, 105	Phthalate ester (plasticizer)
7	9.89	Dimethyl phthalate	195.0648	C_10_H_10_O_4_	135, 133, 120, 105	Phthalate ester (plasticizer)
8	23.40	Pheophorbide A	593.2758	C_35_H_36_N_4_O_5_	533	Chlorophyll degradation product
9	24.99	Pheophytin A	871.5735	C_55_H_74_N_4_O_5_	593, 592, 561, 533	Chlorophyll derivative
10	15.96	2-hydroxy-4-methoxybenzophenone	229.0854	C_14_H_12_O_3_	151, 108, 105	Benzophenone derivative

**Fig. 2:**
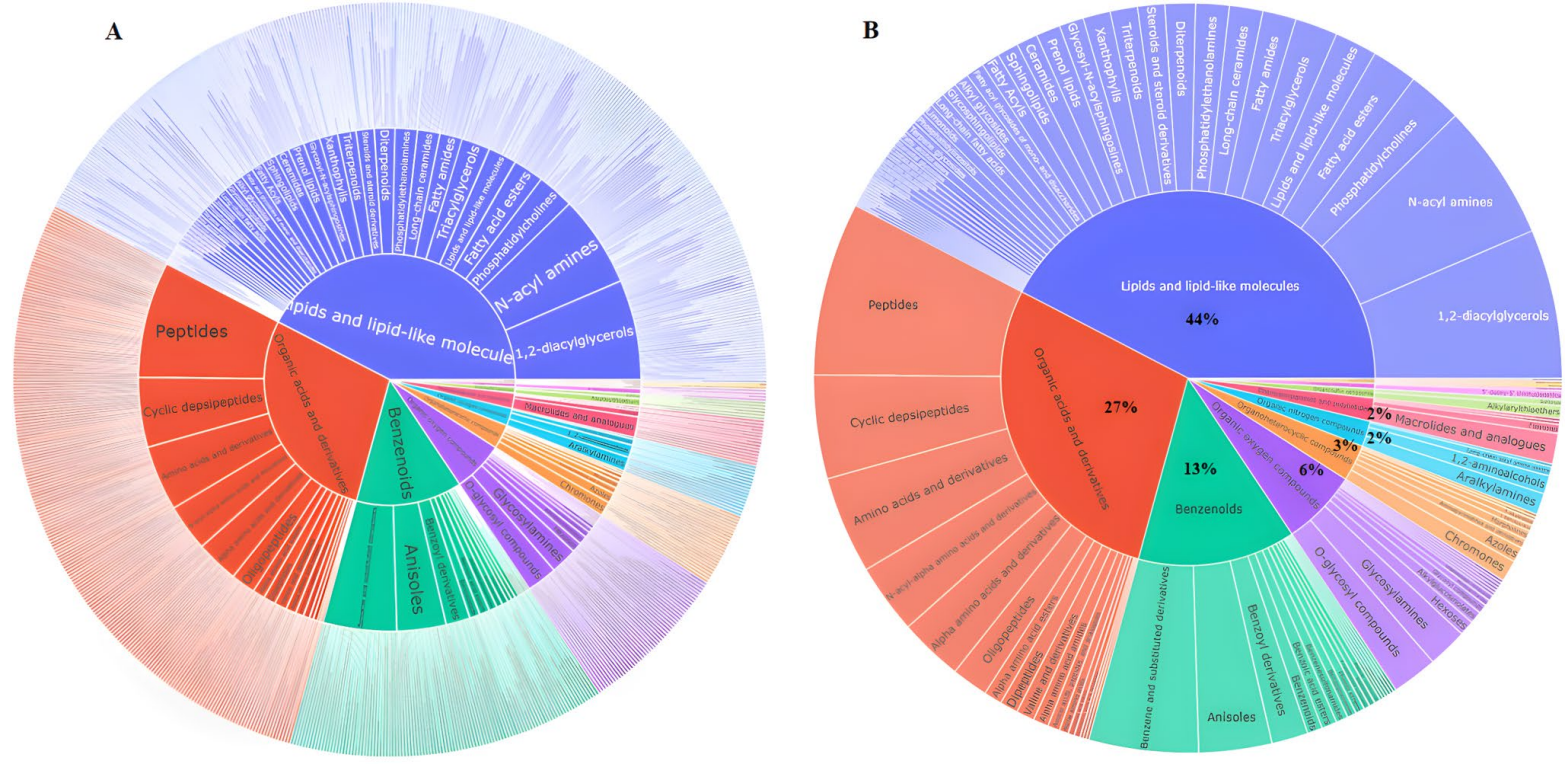
Sunburst plots illustrating the distribution of predicted annotations obtained from SIRIUS for compounds detected in wetland water samples. **A** Putative compound identifications with their corresponding chemical classes and superclasses. **B** Summary of compound distribution based on chemical classes and superclasses only

Due to this complexity, molecular networking revealed numerous self-looped nodes, emphasizing the need to inspect both clustered and unclustered features to fully understand the chemical landscape of the wetland system. The presence of these compounds raises concerns about their potential detrimental effects on freshwater organisms and the broader ecosystem (Levin et al. 2007; Nathan et al. 2024). Although a few compounds were identified, a large proportion of detected features remained unidentified, due to the limited coverage of existing spectral libraries for complex environmental matrices. However, computational tools such as CANOPUS now help bridge this gap by predicting compound classes from fragmentation patterns (Dührkop et al. 2021). According to the metabolomics standard initiative guidelines by Sumner et al. (2007), the assignment of putative compound classes is at a level of 3 confidence. Based on the SIRIUS annotations and CANOPUS class predictions, lipids dominated the chemical profile of water samples, representing 44% of all putatively annotated compounds (Fig. 2). These lipid-related features were most prominent in JHS samples collected after rainfall, as seen in Fig. 1.

### Comparative Differences in Chemical Composition Between Sites Using Molecular Networking

An infographic display of the LC-MS data using molecular networking (Fig. 1) showed that the two sites differ chemically with JHS water samples dominated with lipids, particularly after the rains. JHS, situated approximately 1.5 km upstream of S3 (Figure S1), exhibited a distinct chemical composition, likely influenced by its hydrological position and the unique vegetation and avifaunal communities. These ecological differences may influence each site’s capacity to filter and transform chemical pollutants. Importantly, differences observed in the chemical composition of water before and after rains highlight how hydrological changes directly influence wetland chemistry. Therefore, findings of this study show that both ecology and human activities shape the chemical environment of wetlands.

The dynamic interplay between hydrology and sediment chemistry may explain the differences observed in water chemistry between seasons characterized by stagnant and flowing conditions. Site 3 comprises a valley seep and floodplain, dominated during the wet season by macrophytes and grasses (e.g., *Panicum maximum*, *Digitaria eriantha*, *Sporobolus fimbriatus*) and forbs (e.g., *Cyperus amabilis*, *Bulbostylis burchellii*), with reed beds of *Phragmites australis* and scattered woody vegetation (*Vachellia* spp., *Ziziphus mucronata*). In contrast, JHS is more than 70% covered by *Phragmites australis*, potentially contributing to the observed chemical heterogeneity between the sites.

It is also important to note that the wetland under study is fed by a river catchment that flows through residential settlements, which likely contributes to the chemical pollution of the incoming water. As a result, during periods of high flow or flooding, chemical pollutants from these settlements are transported into the wetland, altering its overall chemical composition as observed in this study. Furthermore, upstream of the wetland, a water treatment plant discharges its effluent into the same river system. Persistent chemical residues from this system are also expected to introduce additional chemical entities into the wetland, further influencing its chemical profile.

There is limited research on wetland chemistry using high-resolution mass spectrometry. Most existing studies have focused on targeted, quantification-based approaches to detect specific chemical contaminants, such as pharmaceuticals (Vazquez-Roig et al. 2011). More recent work used a Q-Exactive™ mass spectrometer operating in full-scan mode, followed by targeted data-independent acquisition (DIA), to detect pharmaceutical and pesticide mixtures in a Mediterranean coastal wetland (Martínez-Megías et al. 2024). To the best of our knowledge, there are no previous studies that have reported the use of untargeted qTOF-MS analysis combined with molecular networking to detect diverse classes of contaminating chemicals in wetlands and to investigate their dynamic shifts in response to seasonal weather changes.

## Conclusion

LC-MS based molecular networking was used for the first in this study to assess the chemical variability of two sites within a natural wetland ecosystem, providing new insights into the ecological functioning of the Nylsvley Ramsar wetland. Findings of this study demonstrated that wetland water chemistry is shaped by various factors such as hydrological dynamics, ecological processes, and anthropogenic activities. The distinct chemical differences observed before and after annual rainfall, particularly for JHS, highlighted the influence of seasonal hydrology in wetland chemistry. Although many compounds remained unidentified due to the limited coverage of spectral libraries for environmental samples, the putatively identified compounds including, pharmaceutical residues, personal care product derivatives and industrial additives, showed the diversity of organic pollutants within the wetland. Notably, chemicals potentially originating from wastewater inputs were also observed, which may be attributed to the wastewater treatment plant located downstream the wetland. The presence of the identified compounds raises concerns about potential toxicological risks they may pose to aquatic species, macrophyte communities and higher trophic levels. However, future studies should quantify these organic pollutants using authentic standards to conduct ecological risk assessment. Moreover, this study highlighted the potential of computational metabolomics tools including, molecular networking and SIRIUS in accelerating environmental monitoring studies.

## Supplementary Information

Supplementary Material 1

## Data Availability

Data will be made available upon request from the authors.
