# Supplementary material for: An LC-MS-Based Molecular Networking Proof-of-Concept for Revealing Ecological Functional Dynamics in Nylsvley Ramsar Wetland Waters
Source: Bull Environ Contam Toxicol. 2026 May 18;116(6):105. doi: 10.1007/s00128-026-04258-3 (PMC13183716; doi:10.1007/s00128-026-04258-3)
Supplement: Supplementary file 1 — Supplementary Material 1 [file 128_2026_4258_MOESM1_ESM.docx]

**Supplementary information**


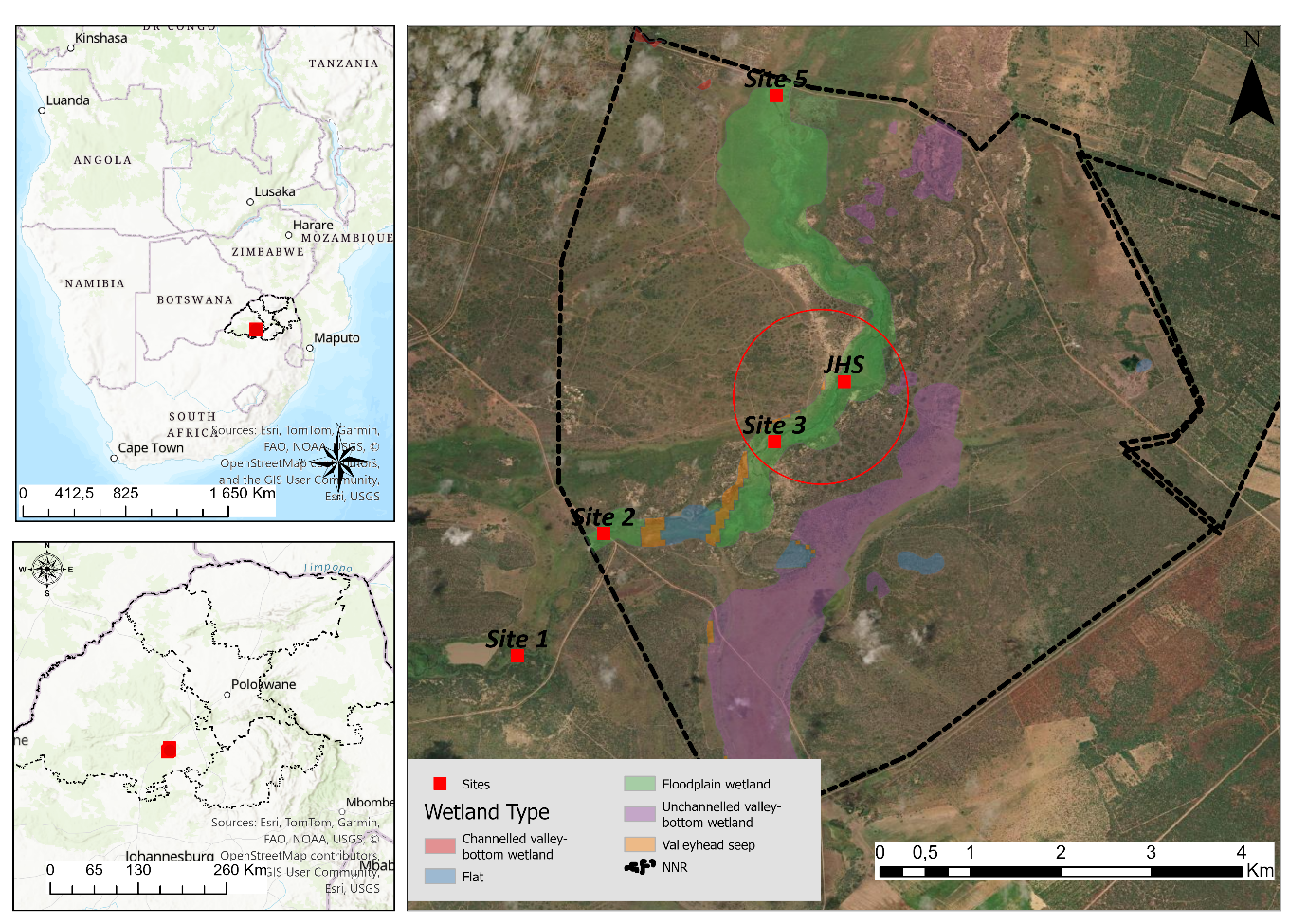


**Figure S1.** Location of study sites within the Nylsvley Nature Reserve in the Modimolle Local Municipality of the Waterberg District, Limpopo Province.


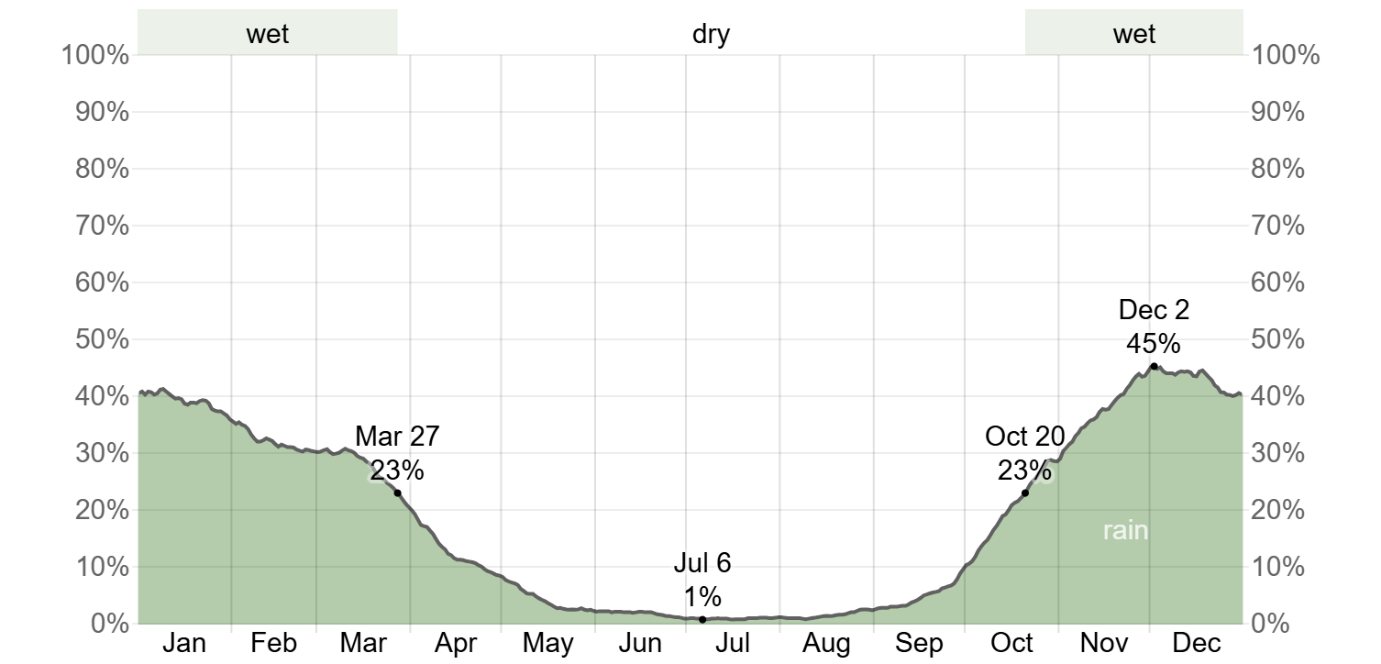


**Figure S2.** Monthly Chance of Precipitation for Modimolle *(WeatherSpark.com, 2025)*


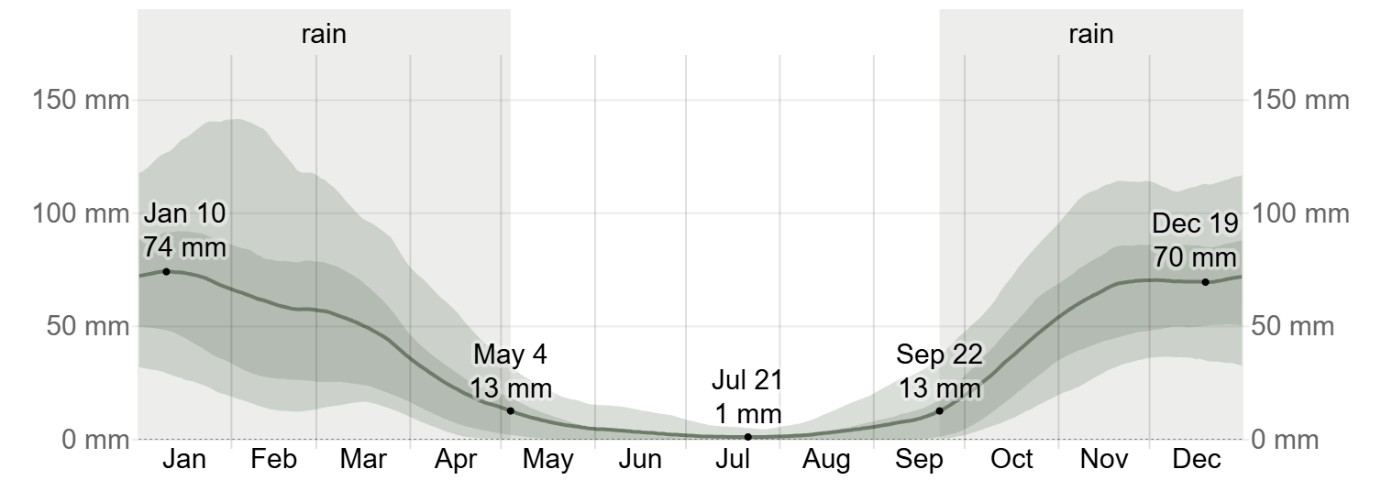


**Figure S3.** Average Monthly Rainfall for Modimolle *(WeatherSpark.com, 2025)*


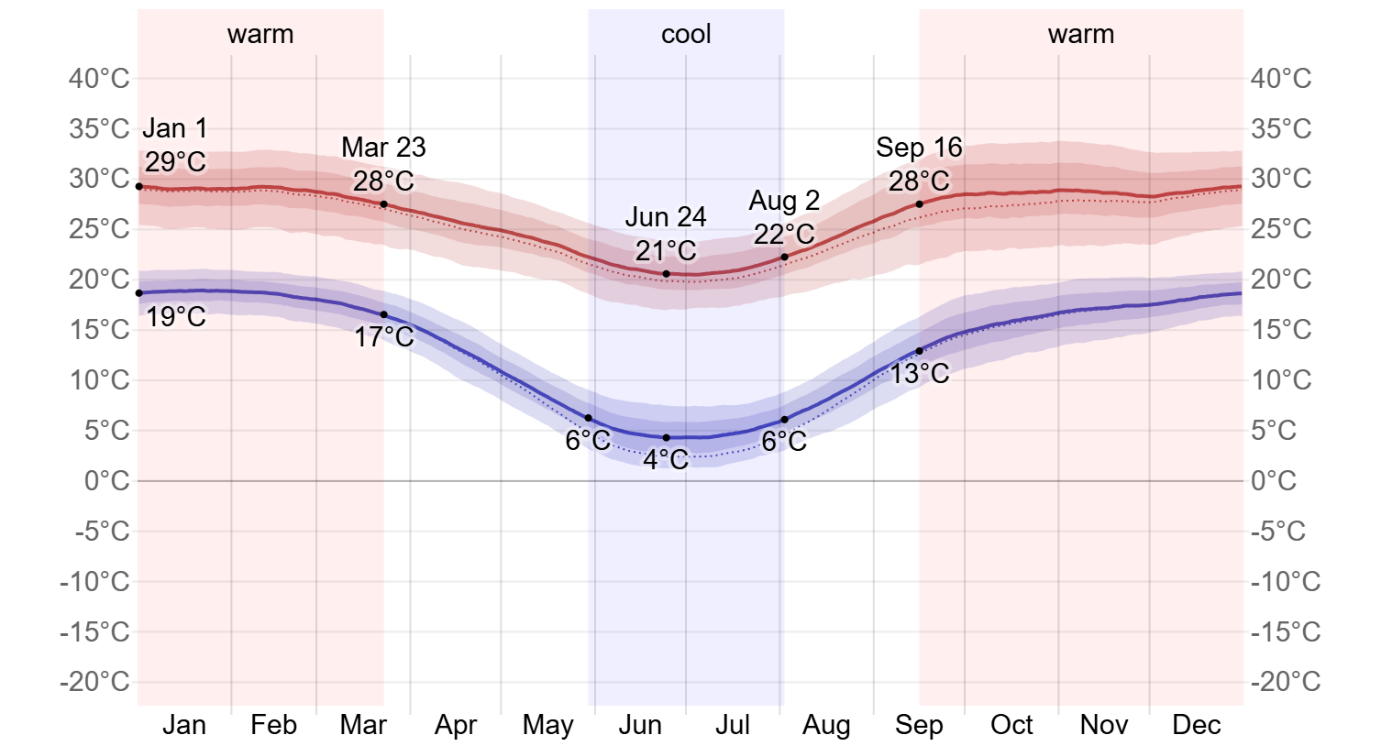


**Figure S4.** Average High and Low Temperature in Modimolle *(WeatherSpark.com, 2025)*

**Feature-based molecular networking link:** bed02c39769446e3971f22a1b1b3a4e0

**Table S1.** Average peak areas of the dereplicated compounds derived from the LC-qTOF-MS data.

|  |  |  | Jacana HIDE | | SiTE 3 | |
| --- | --- | --- | --- | --- | --- | --- |
| # | **Compound name** | **Molecular ion mass** | **After rains** | **Before rains** | **After rains** | **Before rains** |
| 1 | Noscapine | 414.1549 | 3496 | 10641 | 19915 | 5538 |
| 2 | Nicotine | 163.1114 | 7706 | 7853 | 7832 | 8073 |
| 3 | Dextromethorphan | 272.2007 | 252 | 1602 | 3237 | 788 |
| 4 | Erucamide | 338.3419 | 1354 | 1226 | 1106 | 1270 |
| 5 | Oleamide | 282.2785 | 72087 | 57004 | 54824 | 57545 |
| 6 | Monomethyl phthalate | 181.0491 | 2927 | 3191 | 2416 | 3516 |
| 7 | Dimethyl phthalate | 195.0648 | 505 | 769 | 1234 | 1151 |
| 8 | Pheophorbide A | 593.2758 | 1101 | 3045 | 19511 | 642 |
| 9 | Pheophytin A | 871.5735 | 1429 | 1994 | 3719 | 39231 |
| 10 | 2-hydroxy-4-methoxybenzophenone | 229.0854 | 3179 | 2644 | 3008 | 2979 |
